# Management Effects on Gastrointestinal Disease in Red Wolves (*Canis rufus*) Under Human Care: A Retrospective Study

**DOI:** 10.3390/ani14213121

**Published:** 2024-10-30

**Authors:** Amy Clare Fontaine, Jennifer Campbell, Logan Opperman, Larry J. Minter, Karen Wolf, Kadie M. Anderson, Corinne J. Kendall, Emily C. Lynch

**Affiliations:** 1 Department of Biological Sciences, North Carolina State University, Raleigh, NC 27695, USA; amy_clare_f@outlook.com (A.C.F.); jlcampbe@ncsu.edu (J.C.); 2 Department of Statistics, North Carolina State University, Raleigh, NC 27695, USA; lopperm@ncsu.edu; 3 North Carolina Zoo, 4401 Zoo Parkway, Asheboro, NC 27205, USA; jb.minter@nczoo.org (L.J.M.); corinne.kendall@nczoo.org (C.J.K.); 4 Point Defiance Zoo & Aquarium, 5400 N. Pearl Street, Tacoma, WA 98407, USA; karen.wolf@pdza.org (K.W.); kadie.anderson@pdza.org (K.M.A.)

**Keywords:** red wolf, ex situ management, husbandry, gastrointestinal health, diet, kibble, nonsteroidal anti-inflammatory drugs

## Abstract

Red wolves (*Canis rufus*) are the most critically endangered wolf globally, with over 95% of individuals living under human care. Gastrointestinal (GI) disease is one of the leading causes of death among adult red wolves under human care, yet links between management practices and this condition remain unclear. By integrating studbook data, survey-based husbandry information, veterinary records, and necropsy reports, our study explored the relationships between management practices and post-mortem diagnoses of GI mucosal disease among 36 adult red wolves between 2004 and 2022. For final diets fed for a minimum of 4 months before death, we report that wolves fed diets limited to only kibble were more likely to exhibit a greater severity of GI mucosal disease than those fed diets that integrated whole prey or other meat items. In addition, while living at their final facilities for a minimum of 9 months before death, wolves treated with a nonsteroidal anti-inflammatory drug exhibited more severe GI issues than those who were not. Conversely, we found no effect of housing and life history traits on GI health. This study highlights the importance of evaluating management and veterinary protocols on red wolf GI health.

## Simple Summary

Red wolves are critically endangered, and the human-managed population is crucial to the survival of the species. Gastrointestinal (GI) disease is one of the leading causes of death for human-managed, adult red wolves, but the causal factors have not yet been clearly identified. Using life history data, husbandry information, and veterinary records, we examined the relationship between post-mortem, histological GI mucosal disease severity and management strategies. We report that wolves fed a diet that regularly included whole prey or other meat items were less likely to exhibit severe GI mucosal disease compared to those fed a diet composed of only kibble. We also found that wolves treated with nonsteroidal anti-inflammatory drugs were more likely to exhibit more severe GI mucosal disease compared to those that did not receive such treatment. Conversely, we found no effect of housing and life history traits on GI health. This study highlights the importance of evaluating diet and medication choices to enhance individual care in human-managed red wolves.

## 1. Introduction

Red wolves (*Canis rufus*) are the most critically endangered wolf globally, with over 95% of the individuals living under human care [1,2]. Maintaining a healthy population under human care is therefore paramount to the survival of the species. The U.S. Fish and Wildlife Service manages this population for release in collaboration with the Association of Zoos and Aquariums (AZA) as partners in the Red Wolf Saving Animals from Extinction (SAFE) Program [2]. While AZA provides a standardized care manual for red wolf husbandry [3], variation in management is probable within these guidelines, which may, in turn, lead to differential health outcomes [4,5,6].

In human care, gastrointestinal (GI) disease is one of the leading causes of death for adult red wolves. Acton et al. (2000) [7] identified causes of death for 38 captive red wolves older than six months between 1992 and 1996. The authors found that 21% (8/38) of the wolves died of gastrointestinal issues, representing the largest proportion of deaths for this age group. In a later study, Seeley et al. (2016) [8] surveyed necropsy reports from 38 facilities for 175 human-managed red wolves older than six months between 1997 and 2012 and found gastrointestinal disease to be the second most common primary cause of death in gross necropsy (38/175) and histology (24/143) reports for this age group, following neoplasia. Finally, to assess primary causes of mortality, Lynch and Kendall (2023) [5] quantified known causes of death from 33 facilities for 214 captive red wolves born between 2000 and 2020 and reported that 29% of all adult (>=1 year old) deaths within this sample could be attributed to some form of GI disease. Given the significant impact of GI disease on the mortality of the red wolf population, it is critical to understand how management practices and life history traits may influence GI health.

Diet and nutrition may play an important role in the incidence of GI disease within this population. Red wolves in human care receive a variety of food items (e.g., dry extruded kibble, whole prey items, and commercial meat products), provided in varying compositions to create a whole diet that ultimately can impact red wolf health [4,6]. Dry extruded kibble, for example, has been linked to a variety of GI health issues in canids. In domestic dogs (*Canis lupus familiaris*), kibble diets increase the risk of chronic enteropathy [9,10,11] and gastric dilatation volvulus [12]. In addition, kibble diets have been associated with a depleted and imbalanced gut microbiome in domestic dogs [13], Mexican wolves (*Canis lupus baileyi*) [14], and red wolves [4]. Drastic changes to the composition of the gut microbiota can compromise the host’s health, making it prone to disease in general and gastrointestinal health issues more specifically [15].

Housing conditions that may elicit a stress response, such as small enclosure sizes, guest access to an animal’s enclosure, high animal densities, and transfers between facilities, may also significantly impact gastrointestinal (GI) health. Stress-related hormones can bind to receptors in the gut, and chronic stress-induced overactivation of these receptors can result in reduced nutrient absorption, increased mucosal permeability, and immunosuppression [16]. These conditions elevate the risk of gut microbiome destabilization, infection, and inflammation [16]. In certain species of carnivores under human care, enclosure size, guest access, and transfer frequency have been shown to affect both gut health [17,18] and behavioral or hormonal indicators of welfare [19,20,21,22]. High enclosure densities can also adversely affect the gut health of mammals, likely because of interactions between stress-related hormones and the GI immune system [23,24,25].

Life history traits, including age, sex, and genetic profile, could play a role in GI health as well. For example, GI disease or dysbiosis severity has been associated with advanced age [26,27] and inbreeding levels [28] in domestic dogs. Additionally, in some carnivores, severe GI disease while under human care is more common in one sex than the other (i.e., female red pandas [29] and male cheetahs [17]), suggesting possible linkages between sex and GI health.

Finally, veterinary treatment should be considered when examining trends in GI health. Nonsteroidal anti-inflammatory drugs (NSAIDs), while commonly used for pain management and symptoms associated with osteoarthritis [30], can cause adverse effects on GI health [31,32]. In response to gut health issues arising from NSAID use, COX-2 selective inhibitors (e.g., meloxicam and carprofen) were developed to achieve anti-inflammatory benefits while decreasing the risk for GI toxicity [33]. Though successfully utilized in veterinary care, the effects of COX-2 selective inhibitors may still be linked to gut health issues [31,34].

To effectively assess the impact of management practices on red wolf GI health, we completed a retrospective analysis of different diets, housing, veterinary management, and life history traits as potential predictors of the severity of GI mucosal disease in red wolves under human care. Specifically, we aimed to address the following research questions: does the amount of kibble in the diet affect GI mucosal disease severity; do housing conditions (enclosure size, wolf density, guest access, and transfer frequency) affect GI mucosal disease severity; do life history traits (age, sex, and inbreeding coefficient) affect GI mucosal disease severity; and do NSAID prescriptions affect GI mucosal disease severity. Such information could improve the health and welfare of red wolves housed under human management.

## 2. Materials and Methods

### 2.1. Study Subjects and Surveys

Using studbook data [35], we identified wolves that met the following criteria: (1) died at facilities that were likely to maintain thorough necropsy records and submit tissues for analysis (following recommendations from KW, head veterinarian for the Red Wolf SAFE Program), (2) were likely to have maintained a consistent diet of either whole prey, hybrid food items (kibble with whole prey and/or processed meat), or only kibble for more than 50% of their lifetimes (relying on survey data collected from [5]), and (3) were adult wolves (1 year or older) born after 1999, when survivorship stabilizes [5]. This process yielded a candidate pool of 96 wolves from 12 facilities.

A survey (Supplementary File S1) was then emailed to institutional representatives at the 12 facilities, containing questions on diet, housing, and the veterinary histories of the selected wolves. Since we sought to obtain complete nutritional data for each wolf, we also emailed the diet-based questions to an additional facility where 1 of the 96 wolves had spent more than 50% of its lifetime. After a thorough review of records (average response rate across all record requests = 85%, Table 1), we limited our study to wolves with available necropsy results—specifically histopathologic reports that included gastric and/or intestinal tissues that were not too autolyzed to preclude diagnosis—and complete nutritional data. These refined criteria produced a filtered group of 38 wolves.

### 2.2. GI Assessment and Comorbidities

Following previous foundational studies [7,8], we assessed post-mortem histopathological evaluation of the GI tract, as GI biopsies conducted ante-mortem were unavailable. We focused on histological diagnoses specific to the stomach and/or intestinal tract, excluding systemic (whole-body) diagnoses. For each wolf, we recorded the highest disease severity grade assigned by a pathologist to the examined GI tissue(s). Histopathologic severity grades are semiquantitative scores of the relative extent of a particular form of tissue damage (such as ulceration or inflammation) that typically range on a 4- or 5-point scale from normal or minimal to marked or severe [36]. Because the pathologists in our study relied on similar grading terminology across institutions, we created a multi-level term based directly on the GI severity grading used by the pathologists, which included four categories extracted directly from the reports: none, mild, moderate, and marked to severe. Using histopathologic severity grades from different pathologists for statistical analysis can be unreliable due to the potential for subjective grading differences [36]. However, previous research that also used necropsy reports from across institutions has proven valuable to our understanding of GI mucosal disease in red wolves [8]. GI diagnoses that were graded according to severity in this study included inflammatory conditions (i.e., enteritis/gastritis/colitis/inflammatory bowel disease/crypt abscesses), erosion or ulceration, hemorrhage, leiomyositis, and hyperplasia. Wolves that only had GI conditions that could not be graded according to severity (i.e., intestinal lymphoma) were excluded from our analysis, since we were unable to compare the relative severity of these conditions across wolves. This process excluded two wolves, narrowing our sample from 38 wolves to a final group of 36 wolves from 5 facilities.

In addition to GI diseases, we also recorded the other body systems for which histological diagnoses were reported in the final 36 wolves, with the goal of identifying comorbidities. We considered the upper digestive (i.e., the buccal cavity and esophagus) and urinary systems as separate systems from our categorization of GI disease as described above, as our analysis focused on the stomach, small intestine, and large intestine, the most frequent sites of GI pathologies in red wolves [37]. These sites are commonly evaluated during red wolf necropsies and can also be biopsied ante-mortem, making them useful areas to examine for GI disease.

### 2.3. Diet

Three categories were created to describe different diet types. The first category, hybrid diet, consisted primarily of dry kibble, with whole rats or mice offered at least weekly and/or processed meat offered four to six times per week. Since these diet variants all consisted mostly of kibble, but with meat regularly incorporated into the diet (as self-reported by the facilities), we regarded them as a single category based on expected dry matter content. The dry kibble brands and labels used within the hybrid diet category included Hill’s Science Diet Canine Active Adult Chicken, Hill’s Science Diet Canine Maintenance Original Dry, Infinia Turkey and Sweet Potato, Mazuri Exotic Canine, Hill’s Science Diet Active dog food (unspecified label), and one unknown brand. The brands and labels of processed meat used within the hybrid diet included Nebraska Classic Canine, Natural Balance (unspecified label), and Nebraska Canine (unspecified label). The second category, the kibble-only diet, was dry kibble-exclusive, with deer carcasses or parts provided occasionally (on a seasonal basis) or not at all. The dry kibble brands and labels used within the kibble-only diet included Hill’s Science Diet Canine Active Adult Chicken and Mazuri Exotic Canine. The third and final category, the meat-only diet, consisted primarily of meat: whole prey carcasses and parts (elk, deer, salmon, turkey, rabbits, and rodents), with kibble offered on a supplementary basis or not at all. Whole prey items were frozen briefly to kill parasites and then fed either frozen or thawed without added vitamins or supplements, as little to no nutritional value is lost during this process if strict guidelines are followed [38]. The dry kibble brand(s) and label(s) offered supplementarily in this final group were not available. Due to the retrospective nature of our study, we were unable to perform a detailed nutrient analysis comparing the dry kibble and processed meat labels used across facilities based on primary ingredients, as, in multiple cases, the specific labels and nutrient profiles of food items used in previous years were not known.

Using the studbook to track time spent over a lifetime across different institutions, we created three diet predictor terms to test the relationship between diet and GI health: the percentage of the wolf’s lifetime spent at kibble-only facilities, the first diet provided, and the final diet type before death.

### 2.4. Housing

We examined housing conditions at a wolf’s final facility to assess their suitability as predictors of GI disease severity at death. Because extreme stress has been shown to affect gut health in canids within five days [39], it is likely that if housing conditions incur stress, changes to the gut will be apparent in our study group.

First, we established a binary variable to indicate whether a wolf spent more than 50% of its time at the final facility in public view, or “on guest access”, defined as habitats visible to visitors throughout the day or those included in scheduled tours. Next, to examine the effects of wolf density, we created two binary variables representing whether other wolves were within (1) visual proximity or (2) auditory proximity to the subject wolf’s primary enclosure while housed in the final facility. Lastly, we considered the effect of transfers over a lifetime, creating a binary term of never transferred versus 1 or more transfers between facilities.

### 2.5. NSAID Prescriptions and Life History Variables

Prescription of an NSAID at any point in time at a wolf’s final facility was included as a binary term. One wolf was excluded from this analysis due to lack of records. We counted both parenteral and oral prescriptions of NSAIDs, as both administration routes have been correlated with adverse GI effects despite their mechanistic differences [40]. Because both NSAID medications observed in the records (meloxicam and carprofen) are considered selective for COX-2 [41], wolves that received either were combined in our analysis, as their selectivity ratios differ only slightly [42]. As the veterinary record we obtained for each wolf came from its facility of mortality, we were only able to assess the wolves’ prescription histories while housed at these facilities.

The influence of life history variables on GI health was also tested. We explored the inbreeding coefficient for each wolf, as generated by PMx [43], and age at death, following categories included in the most recent population viability analysis for this species [44]. We also included wolf sex as a predictor term.

### 2.6. Analyses

RStudio (version 4.3.3) [45] was used for all analyses. The association() function of the package greybox [46] was used to test correlations across all predictor terms before modeling. Predictors with correlation coefficients of 0.7 or higher were tested separately [47]. In model selection processes using information criteria to assess fit, high correlation coefficients do not affect comparisons of fitness across models if the correlated predictors are included in separate models [48].

Our response variable of GI disease severity was treated as an ordered factor with four levels (none, mild, moderate, and marked to severe), necessitating the use of cumulative link models (CLMs) with a logit link for ordinal logistic regression, using the function clm() from the ordinal package [49].

To test our large number of predictor terms (*n* = 11), we first compared models within each of three categories (diet, housing, and life history/NSAID prescription), including interactions, additive, and single-term models as well as the null model. The Corrected Akaike Information Criterion (AICc) was used to conduct our model selection process, where models with the lowest AICc scores were considered to indicate better-fitting models [50]. Relying on the AICc score to interpret model fit is particularly useful when working with smaller sample sizes with low heterogeneity [51]. When two models with AICc scores within 2 units (the standard penalty of the AICc for adding a term to the model; [52,53]) of each other were competing for the best fit model, the significance of the terms within the model was considered. If the removal of an insignificant predictor did not affect the AICc by more than 2 units, then that predictor was deemed unnecessary for the fit of the model and removed to maintain parsimony [54]. Models with the lowest AICc, after accounting for the significance of the predictors, from each of the three categories were then moved forward into “global models” and, again, we explored the terms as single-term, interactive, and additive. The model from this group of testing with the lowest AICc was considered the best fit.

## 3. Results

### 3.1. GI Assessment and Comorbidities

Our dependent variable categorized the highest GI disease severity score noted for each wolf into the following four levels: none (*n* = 8), mild (*n* = 9), moderate (*n* = 8), and marked to severe (*n* = 11). Thus, over 70% of the sampled wolves (28 of 36) exhibited some degree of GI disease. For these 28 wolves, the diagnoses represented by their highest severity grades were enteritis (*n* = 11), gastritis (*n* = 5), gastroenteritis (*n* = 2), gastroenterocolitis (*n* =1), enterocolitis (*n* = 1), inflammatory bowel disease (*n* = 1), enteric crypt abscesses (*n* = 1), fundic erosion (*n* = 1), gastric hemorrhage (*n* = 1), rectal hemorrhage (*n* = 1), duodenal ulceration (*n* = 1), intestinal leiomyositis (*n* = 1), and ileal hyperplasia (*n* = 1).

For the 11 wolves with marked to severe GI diagnoses, comorbid diagnoses included hepatopancreatobiliary (*n* = 6), integumentary (*n* = 5), endocrine (*n* = 4), renal (*n* = 3), cardiovascular (*n* = 3), respiratory (*n* = 3), lymphatic (*n* = 2), musculoskeletal (*n* = 2), nervous (*n* = 1), urinary (*n* = 1), auditory (*n* = 1), and esophageal (*n* = 1) issues, with only one wolf in this group exhibiting no comorbidities. For reported non-GI histological diagnoses across the other 25 wolves, the primary body systems affected were respiratory (*n* = 14), renal (*n* = 10), reproductive (*n* = 9), hepatopancreatobiliary (*n* = 8), lymphatic (*n* = 8), cardiovascular (*n* = 6), endocrine (*n* = 6), musculoskeletal (*n* = 4), nervous (*n* = 4), integumentary (*n* = 2), urinary (*n* = 1), and salivary (*n* = 1). Only one wolf had no histological diagnoses noted for any body system (GI or otherwise).

### 3.2. Diet

Our additive and interactive diet models included the following fixed effects: final diet (hybrid, *n* =16; kibble-only, *n* =20) and first diet (hybrid, *n* = 9; kibble-only, *n* = 27). The percentage of the lifetime spent at kibble-only facilities (mean = 0.71, min = 0, max = 1.0) was correlated with both final diet (r = 0.79) and first diet (r = 0.87), so these terms were tested separately. From the single-term, additive, and interactive models generated with the three diet predictors, the three best fit models based on lowest computed AICc values were identified (Table 2). The single-term model with only final diet and the additive and interactive models with both final diet and first diet were found to be within two AICc units of each other. Relying then on significance [54], our final model for diet predictors only included final diet as a fixed term (Estimate = 1.5, Std. Error = 0.64, *p* = 0.02).

### 3.3. Housing

The minimum time spent at the final facility was 291 days, approximating 9 months (mean = 2835 days, max = 5981 days). Our full housing model included the following fixed effects: guest access (wolves on guest access, *n* = 5; wolves not on guest access, *n* = 31), transfer history (never transferred, *n* = 29; transferred 1 or more times, *n* = 7), auditory proximity to wolves in other enclosures (yes, *n* = 33; no, *n* = 3), and visual proximity to wolves in other enclosures (yes, *n* = 31; no, *n* = 5). From the single-term, additive, and interactive models generated with those housing predictors, the three best fit models based on the lowest computed AICc values were found (Table 3). Our final housing model only included guest access as the fixed effect, but this term did not yield significance (Estimate = −1.4, Std. Error = 0.86, *p* = 0.1).

### 3.4. NSAID Prescriptions and Life History Variables

Our full life history model included the following fixed effects: age class at death, with ordinal categories of subadult (1–2 years; *n* = 3), adult (2–7 years; *n* = 11), senior (7–10 years; *n* = 8), or geriatric (>10 years; *n* = 14); sex (male, *n* = 15; female, *n* = 21); inbreeding coefficient (min = 0.05, max = 0.1, mean = 0.07); and NSAID prescription at facility of mortality (yes, *n* = 25; no, *n* =10). Wolves who received an NSAID at their final facilities were prescribed either meloxicam only (*n* = 21), carprofen only (*n* = 2), or both at separate points in time (*n* = 2). The average time window between death and the last NSAID prescription received at a wolf’s final facility was 642 days, or 21 months (min = 0 days, the same day as death; max = 2399 days, or about 6 years). For the 24 wolves for which the total number of NSAID prescription events could be determined from the records, the number of prescription events ranged from 1 to 16, with an average of 4 events. For the 94 prescription events (oral prescription events, *n* = 27; parenteral prescription events, *n* = 67) for which treatment durations could be determined from the records, the average treatment duration was 3 days (min = 1 day, max = 14 days).

From the single-term, additive, and interactive models generated with the four life history predictors, we extracted the three best fit models based on the lowest computed AICc values (Table 4). Our final model for life history variables and NSAID use only included NSAID prescription as a single term, though this term did not yield significance in its single-term model (Estimate = 1.35, Std. Error = 0.72, *p* = 0.06).

### 3.5. Global Models

Our full global model included the best-fitting fixed effects identified in our categorical model selection process: final diet, NSAID prescription, and guest access. From the single-term, additive, and interactive models generated with those predictors, we determined the three best-fit models based on the lowest computed AICc values (Table 5). We report our best-fit model for the prediction of GI disease severity scores to be the additive model containing NSAID prescription and final diet.

**Figure 1:**
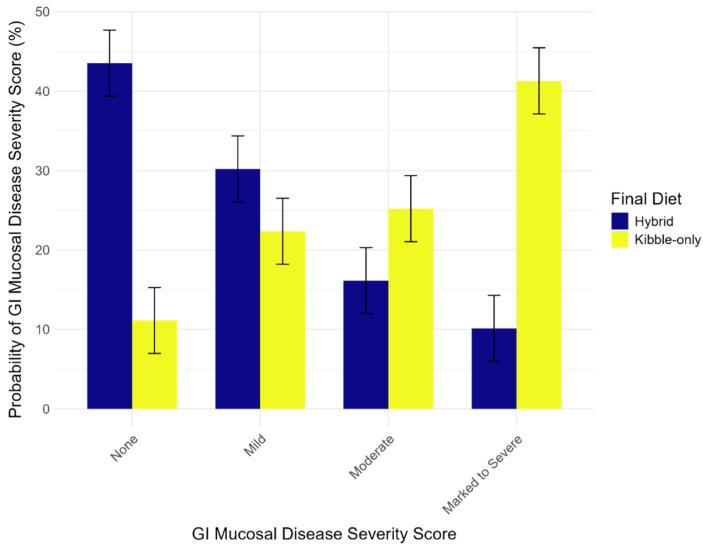
Model results for diet and GI disease. Red wolves fed a hybrid diet were less likely to exhibit severe GI disease compared to those fed a kibble-only diet. Error bars indicate the standard error of the means.

In the best-fit model, kibble-only final diets significantly predicted greater severity of GI disease (Estimate = 2.1, Std. Error = 0.70, *p* = 0.003; Figure 1). For example, for consistent diets prior to death, wolves restricted to a diet of only kibble were 41.30% likely to present a marked to severe GI disease severity score. In contrast, wolves on a hybrid diet were only 10.13% likely to present a marked to severe GI disease severity score.

**Figure 2:**
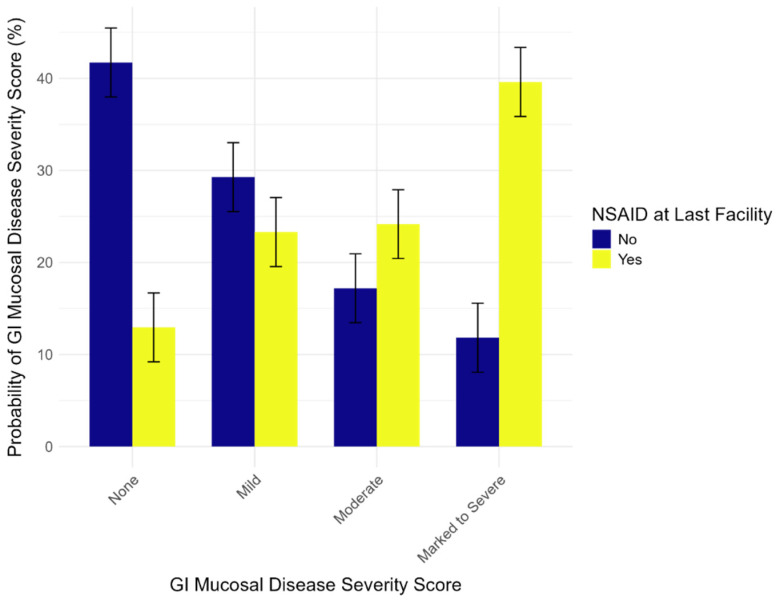
Model results for NSAID use and GI Disease Severity. Red wolves who received NSAID prescriptions at their last facilities were predicted to exhibit more severe GI disease compared to wolves who did not. Error bars indicate the standard error of the means.

We also report that, while housed at their final facilities for an average of 7 years before death, wolves that received at least one NSAID prescription were significantly more likely to exhibit more severe GI disease (Estimate = 1.8, Std. Error = 0.77, *p* = 0.02). For example, wolves administered one or more NSAIDs in this time frame were 27.78% more likely to present a marked to severe GI diagnosis compared to wolves who did not receive the treatment (Figure 2).

## 4. Discussion

This retrospective study investigates the impact of management practices on gastrointestinal (GI) mucosal health in the managed red wolf population. We found that diet and nonsteroidal anti-inflammatory drug (NSAID) use were the strongest predictors of GI mucosal disease severity scores. Given that most red wolves currently live under human care and that GI disease is a leading cause of adult mortality, evaluating management practices that may influence red wolf gut health is crucial for the survival of the species.

We report that kibble-only final diets better predict high severity scores of GI mucosal disease compared to hybrid diets. This finding contributes to the growing literature linking highly processed diets to GI disease among canids (domestic dogs, [9,10,11]; coyotes, [55]). These findings may be rooted in several complications well-known to be associated with kibble diets in canids. First, the processing of kibble can impact gut health. Processing requires heat treatments that aim to improve digestibility while extending shelf life and eliminating pathogens. This process, though, alters the availability of key nutrients [56], modifies levels of microstructure [57], and increases the presence of harmful byproducts [58,59]. In particular, the advanced glycation end products (AGEs) produced during heat processing may instigate pro-inflammatory responses in the GI tract by binding to receptors responsible for upregulating inflammation [60,61]. Dietary AGEs can also increase the permeability of the intestinal epithelium [62], making the gut more vulnerable to ulceration and erosion. It is likely that these effects of heat processing lead to higher GI mucosal disease severity in red wolves on kibble-only diets. Such health outcomes may also be due in part to kibble providing high amounts of carbohydrates while lacking anti-inflammatory micronutrients, leading to greater mucosal permeability and inflammation [63,64]. Highly processed pet foods like kibble are also low in dietary fibers, which are essential to promoting digestive health [65].

Second, an alteration in the structure of the gut microbiome is an emerging pattern linked to GI disease in canids [13,15,66]. Disruptions to the GI microbiome can affect an array of physiological and immunological functions, directly or indirectly [67], and nutrition has the potential to both affect disease conditions and directly change the microbiome [13,68]. Compared to meat-based diets, kibble diets have been directly linked to altered microbiota diversity in the gut in red wolves [6], Mexican wolves [14], and domestic dogs [13,69].

Our findings also suggest a relatively acute onset of diet-based GI symptoms, as the consistent diet given over a minimum of 4 months leading up to death predicted severity of GI issues. Domestic dogs experiencing chronic enteropathy have been shown to respond to dietary changes within 14 days [70], and because the minimum time a wolf was fed a consistent diet before death was 143 days, or approximately 4 months (mean = 2285 days, max = 5854 days), it is probable that the post-mortem histopathology report reflects the last diet type consumed. Red wolves may therefore experience positive outcomes from dietary interventions targeting GI symptoms. In domestic dogs, those with chronic enteropathy and other GI diseases were shown to respond favorably to dietary interventions in about 50% of the cases reviewed by Simpson and Jergens (2011) [71]. Current management techniques already include providing meat to wolves who exhibit symptoms associated with poor gut health (e.g., worsening of body condition, fecal scoring). Certainly, then, gradually increasing the meat content in the diets of red wolves housed in zoological facilities may help mitigate GI health issues. Due to the limitations of the sample size and our reliance on historical records, future work would benefit from incorporating a broader range of wolves, spanning all diet types, to provide additional insights into the predictors and GI health.

Our study revealed that NSAID use predicted GI disease severity. We report that wolves given at least one prescription of NSAIDs within an average of 7 years before death (the time frame for which wolves were housed at their final facilities) exhibited more severe GI mucosal disease upon necropsy than those without NSAID treatment. NSAIDs play a critical role in animal health management due to their anti-inflammatory and analgesic properties and are a powerful tool in promoting animal welfare. It should be noted, though, that these medications generally exert their pharmacological effects by inhibiting cyclooxygenase enzymes involved with prostaglandin production, which can obstruct homeostatic functions, such as gastric epithelial cytoprotection, platelet function homeostasis, and renal blood flow regulation, as well as the inflammatory response, all of which can affect gut health [72,73]. The medications assessed in this study, however, were categorized as selective COX-2 inhibitors, which, generally, have significantly fewer side effects on upper GI health, as they subvert these processes [74]. Therefore, if these medications are affecting gut health in red wolves, the underlying mechanism may not be related to the inhibition of prostaglandin.

Instead, because medications, like nutrition, can influence the composition and activity of the gut microbiota [75], our results could indicate disruptions within the microbiome. Selective NSAIDs, while not directly impacting mucosal gut health as often as traditional NSAIDs, have been demonstrated to adversely affect the gut microbial community [76,77,78]. As previously described, disruptions to the gut microbiome can incite a suite of health issues, including GI disease [15]. Therefore, the increased severity of GI health issues linked with NSAID use in our study may be driven by disturbances in the gut microbiome, especially given that some NSAIDs have been shown to exhibit antibacterial properties [79].

The wolves who received NSAIDs in our study group were prescribed NSAIDs an average of 4 separate times for an average of 3 days at their final facilities, with the last prescription event occurring an average of 21 months before death. This time window between the last prescription event and death, combined with the short average treatment duration, may be interpreted as too wide a gap to directly link NSAID use with gut health issues, as the canid gut can heal as early as 10 days after treatment if mucosal damage occurs [80]. Our finding may then implicate a cascade effect, where at-risk individuals may be more vulnerable to the effects of this medication. Among red wolves, there is evidence suggesting a genetic predisposition within the managed population for IBD [81]. If vulnerable individuals with this predisposition receive NSAID treatment while also exposed to various other environmental factors that can affect gut health, it is then possible that physiological processes responsible for GI disease are exacerbated.

Due to our small sample size, we were unable to distinguish in our analysis between areas within the GI tract that exhibited lesions. NSAIDs are most likely to affect the stomach, so lesions outside of this area would suggest other causes for GI disease [33]. However, if a cascade effect is indeed occurring, we might not expect lesions to be restricted to the stomach following NSAID use. It is also possible that symptoms leading to prescription of NSAIDs may be correlated with GI issues, which could be causing a confounding effect. For example, some studies have shown an association between canine arthritis and gut microbiome composition [82,83], suggesting a potential pre-existing relationship between joint inflammation and gut health. Finally, since histological diagnoses in body systems other than the GI tract were reported for most wolves in this study, comorbidities could be affecting our results. For example, chronic renal disease may adversely impact the composition and functions of the gut microbiota [84]. If a wolf treated with an NSAID at some point in time develops renal disease later in life, it may be difficult to determine whether the presentation of GI disease reflects renal issues, NSAID administration, or both. Thus, further research is needed to illuminate these relationships.

Housing conditions did not predict GI health in our study. Free-ranging red wolves are known to disperse from their natal groups after reaching sexual maturity, at around 2 years old [85]; thus, transfers between institutions may mimic a natural part of the life cycle. Indeed, previous research has not found a correlation between transfer number and survival [5], suggesting this management practice may not induce chronic stress. Wolf density at the facility was also not found to have an impact on GI disease severity. In the wild, reproduction in red wolves declines as population density increases, suggesting that densities and related territorial conflicts are regulated by resource availability [86]. Thus, the presence of additional neighboring individuals in human care may not induce stress because resource availability is not a limiting factor for human-managed animals. Further, while habitats available for public viewing have been found to benefit overall survivorship of red wolves [5], the mechanisms behind this correlation are less clear. Improved monitoring of animals on guest view or more nuanced physiological effects of visitor presence across individuals could explain this relationship [87], which may not directly affect gut health.

Our study found no significant effects of life history variables on GI mucosal disease severity. As previous work did not find sex-biased survival rates or causes of death [5], it is likely that sex does not play a role in GI health in red wolves either. We did not find the inbreeding coefficient to significantly predict the likelihood of GI mucosal disease severity, but it is important to note that this value only reflects the probability of overall relatedness between the sire and dam, rather than similarities at various genomic levels. Further research into the genetic component may be better suited to examine this connection, as some forms of genetic variation have already been shown to predispose wolves to GI disease [81]. Finally, while advanced age and GI mucosal disease severity have been linked in domestic dogs [26,27], we did not find age at death of the individual to be a significant predictor. It may be possible that the lack of association in our study is due to our small sample size, as compared to larger studies in domestic dogs. Another explanation may lie in the significance of diet within this population: perhaps those animals fed a kibble-only diet are more likely to develop severe GI mucosal disease, regardless of age.

## 5. Conclusions

Maintaining the health of the red wolf population under human care is of paramount importance to the survival of the species. With GI disease as one of the primary causes of adult mortality, it is imperative to deepen our understanding of contributing factors to mitigate and improve the health of red wolves. Based on our findings, we can provide several recommendations for the management of red wolves. First, as kibble-only diets predicted GI mucosal disease severity, we encourage the inclusion of more meat-based items in diet planning. Small prey items (rabbit, mouse) to larger carcass feeds (deer) may improve gut health, in addition to retaining more natural foraging and mastication behaviors associated with consumption. Second, careful evaluation of individual health status is encouraged prior to administration of NSAIDs. Assessing stomach condition and considering gastroprotective measures, in particular, may be useful. While the singular causative agent generating GI mucosal disease among red wolves is unknown, it is most likely rooted in complex interactions between the gut microbiome, environmental factors, such as diet, and genetic predisposition [81,88]. Further study of red wolf gut health will continue to improve their management and welfare.

## Figures and Tables

**Table 1:** Response rates for information requested for our study.

Item	Number Requested	Number Received	Response Rate (%)
Husbandry Surveys	13	12	92
Necropsy Reports	96	82	85
Routine Medical Records	96	75	78

**Table 2:** Diet model selection. The top 3 models are presented (K = number of estimated parameters, AICc = corrected Aikake Information Criterion, ΔAICc = AICc difference from the top model, w = AICc weight, and LL = log likelihood. Asterisks indicate significant terms at α = 0.05).

Model	Predictors	K	AICc	ΔAICc	w	LL
1	First Diet + Final Diet *	5	101.94	0.00	0.35	−44.97
2	First Diet × Final Diet *	5	101.94	0.00	0.35	−44.97
3	Final Diet *	4	102.86	0.92	0.22	−46.79

**Table 3:** Housing model selection. The top 3 models are presented (K = number of estimated parameters, AICc = corrected Aikake Information Criterion, ΔAICc = AICc difference from the top model, w = AICc weight, and LL = log likelihood).

Model	Predictors	K	AICc	ΔAICc	w	LL
1	Guest Access	4	105.65	0.00	0.15	−48.18
2	Null	3	105.92	0.27	0.13	−49.58
3	Visual Access	4	106.99	1.34	0.08	−48.85

**Table 4:** Life history/NSAID prescription use model selection. The top 3 ranked models presented (K = number of estimated parameters, AICc = corrected Aikake Information Criterion, ΔAICc = AICc difference from the top model, w = AICc weight, and LL = log likelihood. Asterisks indicate significant terms at α = 0.05).

Model	Predictors	K	AICc	ΔAICc	w	LL
1	NSAID	4	101.75	0.00	0.44	−46.21
2	NSAID * + Inbreeding Coefficient	5	103.84	2.08	0.16	−45.89
3	NSAID + Sex	5	104.42	2.66	0.12	−46.17

**Table 5:** Global model selection. The top 3 ranked models presented (K = number of estimated parameters, AICc = corrected Aikake Information Criterion, ΔAICc = AICc difference from the top model, w = AICc weight, and LL = log likelihood. Asterisks indicate significant terms at α = 0.05).

Model	Predictors	K	AICc	ΔAICc	w	LL
1	Final Diet * + NSAID *	5	94.69	0.00	0.49	−41.31
2	Final Diet * × NSAID *	6	97.01	2.32	0.15	−41.00
3	Final Diet * + Guest Access + NSAID *	6	97.45	2.77	0.12	−41.23

## Data Availability

Data are available upon request from the authors.

## Supplementary Materials

The following supporting information can be downloaded at: https://www.mdpi.com/article/10.3390/ani14213121/s1, Supplementary File S1: Management Survey. Questions from this survey were completed by 12 facilities to provide data on diet, housing, and the veterinary histories of the selected wolves.
