# Supplementary material for: Management Effects on Gastrointestinal Disease in Red Wolves (Canis rufus) Under Human Care: A Retrospective Study
Source: Animals (Basel). 2024 Oct 30;14(21):3121. doi: 10.3390/ani14213121 (PMC11544777; doi:10.3390/ani14213121)
Supplement: Supplementary file 1 [file animals-14-03121-s001.zip › animals-3225977-supplementary.pdf]

Supplementary File S1

We are asking for detailed information on specific deceased wolves who have been identified as valuable individuals for this study:

Studbook #'s:

- Number 1
- Number 2
- etc.

We ask for as much of the following information you are able to provide for each wolf:

1. Health Records
   a. Cause of death
   b. Necropsy records, including histopathology results
   c. All veterinary records (i.e., vaccination records, deworming history (product and frequency), nutrition, stool analysis, body condition, darting/handlings, number of exams and how they were performed, capture protocols, and any other documents available)
   d. Any behavioral information from keeper logs written during wolf's last month of life

2. Housing Details
   a. Enclosure size
   b. Was the wolf on guest access?
   c. Was the wolf in visible proximity to a road?
   d. Was the wolf in visible proximity to wolves in other enclosures?
   e. Approximate distance between enclosures (if in visible proximity)
   f. Was the wolf in auditory proximity to wolves in other enclosures?
   g. Please describe the social composition history of this wolf at your facility over its lifetime, including the last year before death

h. Please tell us the approximate number of wolves housed at your facility during the wolf’s lifetime

3. Diet Details

a. Kibble Brand and Type used while wolf was at your facility (i.e., not just "Taste of the Wild", but "Taste of the Wild Ancient Mountain with Roasted Lamb and Ancient Grains")

b. Whole Prey Details (i.e., species, body parts/organs)

c. Supplementary Items Details (i.e., brand and type of processed meat, fruits, etc.)

d. What percent of the wolf's diet was kibble, what percent was whole prey, and what percent was each supplementary item?
